# Cardiac Pathology in Myotonic Dystrophy Type 1

**DOI:** 10.3390/ijms222111874

**Published:** 2021-11-02

**Authors:** Mani S. Mahadevan, Ramesh S. Yadava, Mahua Mandal

**Affiliations:** Department of Pathology, University of Virginia, Charlottesville, VA 22908, USA; ry3b@virginia.edu (R.S.Y.); mm3zt@virginia.edu (M.M.)

**Keywords:** myotonic dystrophy, RNA toxicity, cardiac pathology, cardiac conduction, triplet repeat mutation, antisense oligonucleotides, fibrosis, fatty infiltration, RNA foci, RNA splicing, sudden death

## Abstract

Myotonic dystrophy type 1 (DM1), the most common muscular dystrophy affecting adults and children, is a multi-systemic disorder affecting skeletal, cardiac, and smooth muscles as well as neurologic, endocrine and other systems. This review is on the cardiac pathology associated with DM1. The heart is one of the primary organs affected in DM1. Cardiac conduction defects are seen in up to 75% of adult DM1 cases and sudden death due to cardiac arrhythmias is one of the most common causes of death in DM1. Unfortunately, the pathogenesis of cardiac manifestations in DM1 is ill defined. In this review, we provide an overview of the history of cardiac studies in DM1, clinical manifestations, and pathology of the heart in DM1. This is followed by a discussion of emerging data about the utility of cardiac magnetic resonance imaging (CMR) as a biomarker for cardiac disease in DM1, and ends with a discussion on models of cardiac RNA toxicity in DM1 and recent clinical guidelines for cardiologic management of individuals with DM1.

## 1. Introduction

Myotonic dystrophy type 1 (DM1) is the most common muscular dystrophy affecting adults and children. DM1 is an autosomal dominant, multi-systemic genetic disorder affecting the skeletal, cardiac and smooth muscles as well as the brain, lens, and endocrine systems [1]. Individuals with DM1 classically have skeletal muscle myotonia, progressive muscle loss and dystrophy, and muscle weakness. Effects on smooth muscles lead to gastrointestinal problems such as swallowing difficulties, bowel dysfunction (constipation and diarrhea), and gallbladder dysfunction (delayed emptying, gallstones) [2].

DM1 is one of the most clinically variable disorders. This is due to its variable age of onset, severity, variable organ involvement and multi-systemic nature. There are four general categories of DM1, based on age of onset: congenital, childhood, adult, and late onset/asymptomatic DM1 [3]. Often, all four categories of DM1 occur in a single family, with increasing severity and earlier age of onset over successive generations. This phenomenon, known as genetic anticipation, was confirmed to be a genuine feature of DM1 by Höweler [4]. The molecular basis for this became apparent with the discovery of the DM1 mutation as an expansion of a (CTG) trinucleotide repeat sequence within the 3′ untranslated region of the DMPK gene [5,6,7]. There is a general correlation between the size of the (CTG) repeat and the age and severity of the disease [8], although this correlation tends to break down when applied to a given individual, likely due to the high somatic instability of the repeats over time and in various tissues. For instance, there are differences between leukocyte (CTG) repeat size and the significantly larger size of repeats in affected tissues such as skeletal muscle [9]. 

## 2. History of Cardiac Disease in DM1

Cardiac manifestations are common in DM1. The combination of myotonia and cardiac manifestations uniquely defines myotonic dystrophies. In his initial description of DM1 as a clinically distinct disorder, Hans Steinert focused primarily on the skeletal muscle aspects of the disorder [10,11]. Notably, Steinert also reported slowing of the pulse (bradycardia) in some cases. In 1911, Griffith wrote the first report focusing on the heart in DM1 where he made note of bradycardia [12]. By the 1920s and 1930s, there were many case reports of cardiac involvement in DM1, and it became clear that the effects on the heart were a common manifestation of DM1 (tabulated in [13,14]). For example, in his survey of 85 case reports, Fisch noted that about 70% had reported abnormal ECG findings, with prolongation of the PR interval being the most common finding. Other reported findings included atrial flutter (Afl) and fibrillation (Afib) as well as bundle branch blocks [14]. Throughout the 1950s and 1960s there were many reports with a variety of cardiac conduction abnormalities in DM1 [15,16,17,18,19,20,21,22,23,24,25]. Conduction defects were often noted to have preceded overt skeletal muscle effects. Sudden death was also noted, and several studies also reported ventricular tachyarrhythmias and complete atrioventricular (AV) dissociation (two conduction disturbances often associated with sudden death). More recent natural history studies including a 13-year follow-up study of 83 patients with DM1 have noted that though there is a correlation between severity of skeletal muscle involvement and cardiac conduction defects in some patients; in many others, cardiac disease progression was more rapid than skeletal muscle disease [26].

## 3. ECG and Conduction Defects in DM1

The most common ECG abnormalities reported in DM1 patients is prolongation of the PR interval (>200 ms), also called first degree atrioventricular block (AV block). It is seen in 25 to 45% of patients in a cross section of primary and meta-analyses studies [27,28,29,30,31]. Other ECG abnormalities include bundle branch blocks and prolongation of the QRS interval (>120 ms) which occur in an additional 15–20% of cases. Clinically, the individual may most often be asymptomatic, or may experience slow heart rate, dizziness, palpitations, lightheadedness, fatigue, or sometimes syncope.

Another common arrhythmia is atrial fibrillation/flutter (AFib/Afl) which occurs in different studies at frequency ranging from 5–30% of cases. Studies of adult DM1 patients, using 24-h Holter monitoring (i.e., ambulatory monitoring), reported an 8 to 22% incidence of Afib/Afl in patients with severe DM1 [32,33]. Similar findings were reported using standard 12 lead ECGs [34]. A literature review of myopathies, covering 1966 to 1987, found an incidence of over 30% for Afib/Afl in DM1 patients [35], while a recent literature review of DM1 literature by Russo covering 2002–2020 reported an incidence of about 11% [36]. Similar results were seen in smaller cohorts of DM1 patients [37,38]. A recent prospective study over two years of 70 patients with DM1, found Afib/Afl developed in 26%, with evidence of interatrial block in more than 30% of the patients. In a study of 161 DM1 patients (most of whom were asymptomatic) followed prospectively for up to 25 years, the authors found about 17% developed Afib/Afl and found it to be an independent predictor of death in DM1 [39]. Groh et al. also found Afib/Afl to be an independent predictor of sudden death in DM1 [27].

A recent, intriguing study found that increased levels of serum NT-proBNP and copeptin (a C-terminus peptide generated from pre pro-peptide of arginine vasopressin), two tests used in monitoring heart failure, might be predictors of Afib onset in a DM1 population, suggesting a potential for identifying patients requiring more frequent monitoring and follow-up [40]. Other groups have also reported that elevated NT-pro-BNP levels are an independent predictor of cardiac conduction abnormalities [41].

Though DM1 cardiac conduction defects are classically reported in adult patients, cardiac arrhythmias are often found in young patients, including adolescents with a history of congenital DM1 [42,43,44,45]. Several studies have also found positive correlations between the CTG repeat size (in blood) and severity/or presence of cardiac conduction defects, and risk of sudden death in DM1 patients [28,46,47,48], while others have found no such correlation [49]. 

Early mortality is an unfortunate outcome of DM1 with a mean age of death around 50–55 years of age [29,50]. Death from progressive respiratory failure is the most common cause reported in about 40% of these cases [27,29,50]. This is closely followed by sudden death due to presumably cardiac causes, in about 30 to 40% of cases [27,50,51]. Limited data exists on the nature of the cardiac rhythm at time of sudden death. Not surprisingly, ventricular tachycardia, complete heart block, asystole, and electromechanical dissociation have been reported in the few monitored cases [27,29,50,51]. In most cases, the cause of sudden death is unclear, and sudden death also occurs in patients with no antecedent history of cardiac issues. Various large-scale studies have reported correlations between the presence of conduction defects, pacemaker implantation, age, size of the CTG repeat expansion, atrial arrhythmias, family history and male sex and the risk of sudden death [27,28,29,51,52,53]. In contrast, other studies have reported no correlations between CTG repeat size and sudden death [54].

## 4. Pathology of the Heart in DM1

For the first half of the twentieth century, there were few if any histological studies of hearts from individuals with DM1 (reviewed by Fisch [14]). The first detailed autopsy study was from a 41-year-old DM1 patient with a history of atrial flutter who died suddenly [55]. Of note, histological evaluation found moderate fatty infiltration of the atrial myocardium and diffuse fibrotic changes in the ventricular myocardium, with variably sized and hypertrophied myocardial cells. Notably, the heart was not enlarged and there was no coronary vessel disease. A similar finding of interstitial myocardial and subendocardial fibrosis was noted in another study of that time [18]. Franks reported a case with similar findings and additionally noted degeneration and fatty infiltration in the AV node in another case report [56]. Light and electron microscopic studies reported on interstitial fatty and fibrosis distributed throughout the heart and myocyte hypertrophy and degeneration [57,58,59].

In 1988, Nguyen published the most comprehensive study on the pathology of the heart in individuals with DM1 [60]. In this study of 12 autopsy cases, the authors found evidence for fibrosis and fatty infiltration of the conduction system (SA node, AV node, bundles of His) and ventricular myocardial fibrosis in almost all the cases. This was associated with myocyte hypertrophy and disarray. In another study of 10 DM1 patients in whom endomyocardial biopsies were obtained, the authors found similar evidence of myocardial fibrosis and fatty infiltration with hypertrophic myocytes in most cases, with some showing evidence of mild myocarditis [61]. Notably, most of these patients were asymptomatic and had only mild or no ECG changes and normal echocardiograms. Thus, it was evident that myocardial pathological changes preceded clinical sequelae or ECG changes.

**Figure 1:**
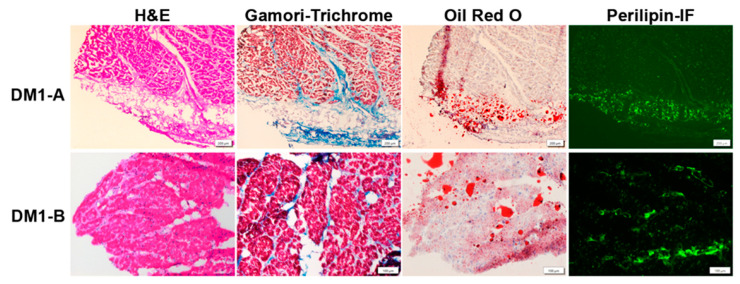
Cardiac Pathology in DM1. DM1-A (subendocardium) and DM1-B (myocardium) are autopsy specimens with evidence of typical cardiac pathology in DM1 showing interstitial fibrosis and fatty infiltration. H&E staining shows interstitial fibrosis (light pink regions) between cardiomyocytes as well as fatty infiltration (clear circumscribed areas in the middle of the tissue indicative of fat droplets). Gamori-trichrome staining highlights collagen (blue). Oil Red O staining detects lipids (red). Perilipin immunofluorescence (Perilipin-IF) (green) detects perilipin, a surface marker on fat droplets. Scale bar is 200 µm for above row and 100 µm for bottom row.

There are some recent studies with good histologic examples of cardiac pathology [62,63]. Figure 1 shows examples of cardiac pathology in DM1.

## 5. Molecular Markers of DM1 in the Heart 

**Figure 2:**
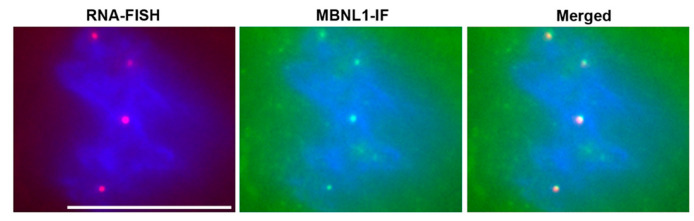
RNA Foci in DM1 Cardiomyocytes. RNA-FISH (RNA fluorescence in-situ hybridization) with a CY3 labeled (CAG)_10_ probe detects nuclear foci of mutant *DMPK* mRNA (red dots). Immunofluorescence microscopy for MBNL1 (MBNL1-IF) shows sequestration of MBNL1 (green dots). Merged image shows co-localization of RNA foci with MBNL1. Nuclei are stained blue with DAPI. Scale bar is 10 µm.

Soon after the discovery of the DM1 mutation, the Singer lab reported that the mutant RNA forms nuclear RNA foci in DM1 cells and skeletal muscles [64]. RNA foci were subsequently reported to be present in DM1 cardiac muscle [65], and in mouse models of RNA toxicity [66]. The RNA-binding protein muscleblind-like splicing regulator-1 (MBNL1) co-localizes with the RNA foci in cardiac tissues. Now, the presence of RNA foci in tissues is a hallmark of DM1 pathology (Figure 2). In addition to RNA foci, the most notable molecular change associated with RNA toxicity in DM1 is the numerous splicing changes that occur. Hundreds of splicing changes occur, whereby there are disproportionately more embryonic isoforms expressed in adult tissues or cardiomyocytes [65,67,68,69]. However, it is not clear which of these changes are causing the cardiac phenotypes in DM1. 

One of the most mentioned splicing defects is that of *SCN5A*, which encodes the α-subunit of the cardiac voltage-gated Na^+^ channel, Na_v_1.5. In humans, a variety of missense, nonsense, splice site, and deletion/duplication mutations occurring as dominant mutations in *SCN5A* result in gain or loss of function and cause a variety of cardiac conduction abnormalities [70]. These conduction abnormalities have a significant overlap with those found in individuals with DM1. In DM1, the splicing switch in *SCN5A* transcripts from the adult (with exon 6B) to the fetal isoform (with exon 6A) results in a higher proportion of *SCN5A*-exon 6A transcripts. The fetal isoform has slower kinetics of activation/inactivation and lower depolarized threshold for activation [71]. Forced switching from adult to fetal isoforms by treating newborn wildtype mice, using viral delivery of antisense RNAs designed to mask splice sites, led to a variety of cardiac conduction phenotypes reminiscent of those seen in DM1 [69]. A proportion of mice at four to six months of age displayed slight prolongation of the PR interval on ECGs, atrial fibrillation, mild cardiac fibrosis, and sudden death [69]. As the authors stated, though this study shows the potential for altered *SCN5A* splicing to cause some DM1 related cardiac phenotypes, the phenotypes were relatively mild compared to that seen in DM1 patients. Another study used CRISPR/Cas9 to delete *Scn5a* exon 6B resulting in exclusive expression of the fetal *Scn5a* exon 6A isoform in a mouse model [72]. This resulted in alteration of the exon6A/exon 6B ratio from 10% exon 6A in wildtype mice, to about 70% exon 6A in heterozygote (*Scn5a*^Δ*e6B/+*^) mice and 100% exon 6A in homozygote (*Scn5a*^Δ*e6B/*Δ*e6B*^) mice. As the authors stated, this alteration is much greater in comparison to the 35% exon 6A that the authors found using hearts from DM1 patients. They were able to phenocopy the results from the study of Freyermuth et al. [69]. Of note, these studies did not address the contribution of this splicing abnormality in the context of RNA toxicity. Thus, it is still uncertain as to which of the plethora of splicing defects is relevant to the cardiac phenotypes in DM1. Admittedly, it will be a difficult task to dissect their individual contributions. 

## 6. Imaging Studies and the DM1 Heart

In clinical practice, ultrasound echocardiography is used routinely to assess cardiac anatomy and function. Many studies have been published of echocardiography in DM1 patients. From the earliest studies [73], most of them found minor changes and typically found no significant adverse changes in left ventricular systolic or diastolic function or cardiac ejection fraction [74,75,76]. Some reports mention mitral valve prolapse [75,77,78,79]. Many reports found wall motion abnormalities, decreased myocardial velocities and diastolic dysfunction, often in the absence of any overt cardiac disease or reductions in ejection fraction [77,78,80,81,82,83,84,85].

The last decade has seen multiple studies documenting cardiac magnetic resonance imaging (CMR) changes in the heart of individuals with DM1. Many investigators commented on the frequency of cardiac fibrosis and the possibility that CMR could be a valuable biomarker for DM1 [86,87,88,89,90,91,92,93,94]. CMR can be used to assess the structural and functional parameters seen by echocardiography, and in addition, it provides a means for assessing fatty infiltration, edema and diffuse and focal fibrosis. However, CMR is much more expensive, requires more expertise, and not standardized as well as echocardiography.

DM1 cardiac pathology is often associated with fibrotic/adipogenic changes. Clinical detection requires invasive endomyocardial biopsies, or autopsy evaluation. ECG changes associated with the cardiac pathology are typically related to large patches of localized fibrosis/adiposis. One of the key advantages of CMR is the potential to detect changes earlier. Various CMR parameters are reported to change in DM1 hearts, including increased T1 relaxation times, increased extracellular volume (ECV) fraction, decreased strain measurements, and increased late gadolinium enhancement (LGE).

Overviews of CMR can be found in reviews of clinical and pre-clinical CMR [95,96,97,98,99,100]. Briefly, T1 mapping measures the spin-lattice relaxation time to re-equilibration after magnetization of protons in a tissue. Clinically, native T1 mapping (pre-gadolinium imaging) is a composite of signals from cardiomyocytes and the extracelluar volume (ECV) and is influenced by edema and increased interstitial space (e.g., fibrosis, protein deposits). Increased T1 relaxation times are sensitive, reproducible, and quantifiable indicators of these pathologic changes, and can detect more diffuse extracellular changes. LGE is the reference method for imaging focal myocardial fibrosis and is detected by reduced T1 times in scarred tissues that retain the gadolinium, resulting in hyper-dense images. ECV is a calculated value based on T1 measurements (pre and post gadolinium) that is corrected for the blood pool (via hematocrit measurements) and that, when increased, is a strong indicator of interstitial or replacement fibrosis and edema in the heart. Strain measurements inform about the degree of deformation (e.g., contraction) of a myocardial segment and are typically expressed as a percentage change.

In one of the earliest CMR studies in a cohort of 14 DM1 patients, they detected abnormalities in 11 patients, indicative of myocardial fatty infiltration and fibrosis [101]. A study of 43 DM1 patients found that CMR evidence of diffuse fibrosis correlated with widened QRS intervals on ECGs [102]. In a 2012 study of 33 DM1 patients, Turkbey et al. found multiple alterations in CMR including increased T1 values that correlated with changes in longitudinal ECGs, and shortened T1 values reflecting the presence of diffuse myocardial fibrosis [86]. Another study of 80 patients found CMR abnormalities in 35 of them (>40% of patients), with evidence of left ventricular (LV) systolic dysfunction and focal fibrosis that associated with ECG abnormalities [92]. A similar incidence of cardiac fibrosis (40%) with contrast enhanced CMR was seen in a study by Petri et al. [90]. In a 2019 study, Chimelewski et al. reported about 42% of patients with abnormal CMRs with multiple parameters indicative of fibrosis (diffuse and focal), decreased strain and LV-ejection fraction, and found increased prevalence of ECG changes (especially Afib/Afl) in patients with abnormal CMR studies. Cardona et al. also found about 40% of patients had abnormal CMR exams with evidence of increased fibrosis, but did not find a correlation with surface ECG findings, again emphasizing the point that CMR evidence of cardiac fibrosis occurred in the absence of standard ECG changes [88]. Luetkens et al. studied a cohort of DM1 patients without known cardiovascular disease and found CMR evidence of diffuse fibrosis (i.e., increased T1 and ECV values; patchy and extensive increased LGE uptake) in about 35–40% of patients, decreased LV-ejection fraction, and decreased myocardial strain measurements [91]. Similar findings of increased ECV and decreased cardiac strain were reported in a smaller cohort of DM1 patients without evidence of cardiac disease [87].

Thus, there is a growing body of evidence supporting the utility of CMR as a clinical biomarker for cardiac disease in DM. Multiple parameters are ascertained, that might be useful in clinical trials. Furthermore, CMR detects subclinical disease (i.e., fibrosis, fatty infiltration, edema, myocardial wall motion abnormalities and dyskinesis) that could portend worsening heart disease and may help in predicting those at risk for sudden death. Implementation is challenging due to the expense, expertise, standardization required for CMR, and clinical challenges of performing CMR in patients with limited mobility and respiratory issues. However, this is changing with the increased number of studies, increased familiarity with DM1 patient needs at specialized centers, and more routine acceptance and use of the technology in clinical practice.

## 7. Cell and Drosophila Model Systems for Studying Cardiac RNA Toxicity in DM1

A number of studies have used cell culture and model organisms to study aspects of DM1 cardiac pathology. We will discuss only a few of the more recent ones. Recently, DM1-induced pluripotent stem cells (iPSCs) have been used to generate cardiomyocyte-like cells [103,104]. The study by Poulin et al. used a normal iPSC cell line and two DM1-iPSC cell lines differentiated to cardiomyocyte lineage to identify several DM1 related splicing defects in DM1 iPSC derived cardiomyocytes, and the investigators also demonstrated abnormal ion channel functions by cellular electrophysiology studies, including changes in Na_v_1.5 gating and slower conduction veloicites [104]. The study by Dastidar et al. used CRISPR/Cas9 to excise the (CTG) repeat sequence from DM1 iPSCs and used transcriptome analyses and cell-based contractility studies to compare the same DM1 cells before and after excision of the (CTG) repeats [103]. They found significant DM1 related differences in gene expression, splicing patterns and conduction velocities between the original DM1 cells and the ones with excised (CTG) repeats. These studies show the potential utility of iPSC-based approaches to study the cellular basis of RNA toxicity in the heart, and the possibility of their utility in cell-based drug screens to identify potential therapeutics for cardiac complications of DM1.

Drosophila models have been used extensively, primarily by Artero and Llamusi labs, to study cardiac aspects of RNA toxicity in DM1 [105,106,107,108]. These models express long (CUG) RNAs in the heart, resulting in irregularities in cardiac rhythm, contractility, and reduced lifespan. The fruit flies were used to test candidate small molecules such as pentamidine and daunorubicin which disrupt MBNL1 binding to (CUG) repeats, and which were shown to have beneficial effects in these models. Another group has used cardiac specific depletion of MBNL1 (Mbl) or overexpression of CELF1 (Bruno-3) in Drosophila to model cardiac conduction defects, and to identify potential new targets such as *CACNA2D3* (a regulatory subunit of Ca-α1D/Ca_v_1.2 voltage gated calcium channel) [109]. As with the cell-based assays, these studies show the potential of these models to study mechanisms of RNA toxicity, and in drug screens designed to identify therapies for DM1.

## 8. Mouse Models of RNA Toxicity in DM1 with Cardiac Phenotypes

In 2006, we published the first report of a mouse model of RNA toxicity in DM1, which had cardiac effects [66]. In this inducible model, the *DMPK* 3′UTR is expressed as part of a GFP transcript that is driven by the human *DMPK* promoter. Surprisingly, these mice developed classic DM1 phenotypes in skeletal muscle and heart despite expressing a normal *DMPK* 3′UTR with (CUG)_5_. Though these mice did not have any RNA foci, the transgene transcript was found to interact with MBNL1 [110]. Of note, these mice developed a variety of cardiac conduction defects ranging from prolongation of the PR interval (first degree heart block) to complete heart block and sudden death [66]. Importantly, silencing transgene expression reversed the cardiac conduction abnormalities and myotonia, thus providing the first in vivo proof of principle for therapeutic strategies aimed at treating DM1 by silencing expression of, or getting rid of, the mutant *DMPK* mRNA [66].

As mentioned in the description of the first DM200 lines [66], those mice in the heterozygous state lacked DM1 related phenotypes despite having RNA foci (likely due to low transgene expression) [66]. Over the past decade, we have subsequently developed a better DM200 mouse model by making new transgenic mice, breeding them to homozygosity and interbreeding lines, to generate a new DM200 model which expresses mRNAs containing the *DMPK* 3′UTR with (CUG)_200_ at a high enough level when induced, to have myotonia, RNA foci and cardiac conduction defects [111]. These new transgenic mice use the same transgene design as for the (CTG)_5_ mice [66], but with a (CTG)_200_ tract. Upon transgene induction, these mice develop a variety of cardiac conduction defects including first degree heart block, atrial fibrillation, increasing degrees of atrioventricular conduction defects, complete heart block and sudden death. They also exhibit RNA splicing defects in the heart, decreased expression of connexin proteins, and cardiac fibrosis. Using these mice, we found for the first time that antisense oligonucleotides (ASOs) could treat cardiac pathology in DM1 [111].

The Cooper lab subsequently produced an inducible mouse model of cardiac specific RNA toxicity in 2007 [112]. This model had an inducible transgene expressing exon 15 of *DMPK* with 960 interrupted CUGs. These mice exhibited a severe phenotype and died within two weeks, exhibiting dilated cardiomyopathy, some areas of focal fibrosis and hypertrophic cardiomyocytes. Progressive lengthening of the PR intervals and QRS complexes were seen within two days of transgene induction followed by progressive heart block. Echocardiography showed evidence of systolic and diastolic dysfunction. There were RNA foci sequestering MBNL1, increased CELF1, and splicing defects in the hearts. Unfortunately, this model was not sustainable (communication with Dr. Cooper). 

Recently, another inducible/reversible mouse model of RNA toxicity in DM1 (TREDT960I) was reported from the Cooper lab, with cardiac specific expression of an RNA with exons 11–15 of *DMPK* with 960 interrupted CUGs [113]. These mice show RNA foci and a multitude of RNA splicing defects, decreased expression of connexin-40, and had prolongation of the QRS and QT_c_ intervals, but did not show the prolongation of the PR interval, progressive heart block or sudden death seen in individuals with DM1. They did display atrial arrhythmias spontaneously and especially with intracardiac pacing induction. These phenotypes were reversible with cessation of induced transgene expression.

Another recent mouse model of RNA toxicity in DM1 is the LC15 mouse with ubiquitous expression of a luciferase mRNA with an expanded *DMPK* 3′UTR (CTG)_250–400_ [114]. Unfortunately, the expression level was not high enough to elicit myotonia or splicing defects in skeletal muscles. However, RNA foci and splicing defects were present in the hearts of these mice. These mice displayed slight prolongation of the QT_c_ interval and widening of the QRS interval on ECGs but no cardiac conduction defects, and showed no evidence of cardiomyopathy by echocardiography in one year old mice.

The most commonly used mouse model of RNA toxicity, the HSA-LR mouse is a skeletal muscle specific model [115] and thus is not relevant to studies of RNA toxicity in the heart. The other commonly used mouse model is the DMSXL which expresses the human *DMPK* transcript with (CUG)_≥1000_ under the regulatory control of the human gene locus [116]. These mice have RNA foci and a few, mild splicing defects in the heart. Baseline ECGs in these mice showed no obvious abnormalities. However, when flecainide, a class-I-antiarrhythmic drug that blocks sodium channels, was used in eight-month-old DMSXL mice, it induced ECG changes such as prolonged PR intervals and higher degree heart block. Echocardiography did not differ from wildtype mice and histological studies did not find evidence of fibrotic disease [117]. Table 1 summarizes the mouse models of RNA toxicity.

A number of mouse models have been generated in order to understand the molecular mechanisms of DM1. Initial reports suggested DMPK haploinsufficiency could lead to conduction abnormalities in mice [118]. However, conduction defects were not reported in another mouse model of DMPK deficiency [119]. There were concerns that since ASOs targeting the *DMPK* transcript affected both mutant and wildtype *DMPK* mRNAs, that the treatment could lead to adverse cardiac effects. However, a recent study of DMPK deficient mice found no evidence of conduction abnormalities on ECGs for up to 18 months of age [120]. Our own unpublished data confirms the results from Carrell et al. [120]. Furthermore, ASO treatment of haploinsufficient DMPK mice [120] and RNA toxicity mice with conduction defects (i.e., DM200 mice) [111], led to significant reductions in *Dmpk* mRNA levels without adverse effects on cardiac conduction.

Most models of RNA toxicity in DM1 involve MBNL sequestration leading to functional deficiency and increased CELF1 activity, reviewed in Braz et al. [121,122,123,124,125]. Mouse models show that increased expression of CELF1 [122] and compound loss of MBNL proteins [123,124] or loss of MBNL1 [125] can lead to cardiac manifestations. Mice with a four- to eight-fold increase in CELF1 levels in the heart died within two weeks from severe cardiac disease due to dilated cardiomyopathy and degeneration of cardiomyocytes. They also exhibited RNA splicing defects and cardiac conduction abnormalities (increased PR intervals and widened QRS complexes). Unfortunately, the severity of the phenotype precluded further studies in these mice. However, neither the rapidity and severity of disease in these mice, nor the levels of increased CELF1, are typical of cardiac disease in DM1 patients. The data on cardiac effects of MBNL1 deficiency is conflicting. The MBNL1 knockout model published by Dixon et al. had decreased viability, sudden death, multiple DM1 relevant cardiac phenotypes including conduction abnormalities, widened PR intervals, interstitial myocardial fibrosis, and RNA splicing defects [125]. However, the MBNL1 knockout model reported by Kanadia [126] does not show these effects, and requires the additional partial loss of MBNL2 to bring out cardiac phenotypes [123]. Since the design of the Dixon and Kanadia models was identical, it was proposed that the differences in genetic background (and thus potential modifier genes) could account for the different phenotypes [125].

More recently, the RNA binding protein RBFOX2 was found to be significantly increased in hearts from individuals with DM1, and this was associated with an RNA splicing shift in *RBFOX2* mRNAs to a non-muscle isoform [127]. The authors showed that this may be related to miRNA deregulation, a mechanism which has been previously proposed to play a role in cardiac pathology in DM1 [128,129,130]. Mice with overexpression of CELF1 in the hearts also showed this splicing switch in *Rbfox2*. Overexpression of this non-muscle isoform in mice resulted in sudden death, and abnormal ECG changes such as prolongation of the PR interval, prolonged QT intervals, sinus pauses and premature ventricular contractions. However, there was no evidence of fibrosis or histologic abnormalities in the hearts of these mice. There were significant and numerous transcriptomic changes in splicing and gene expression that were recapitulated in DM1 hearts. Thus, there is significant evidence to suggest that mis-regulation of RNA binding proteins related to DM1 pathogenesis results in cardiac pathology that phenocopies many aspects seen in DM1.

## 9. Clinical Care Guidelines for the Management of Cardiac Issues in DM1

It is essential for individuals with DM1 to have a careful evaluation of their cardiac status and to have regular surveillance throughout their lives. Given the high incidence of cardiac disease (up to 75% of patients) and the risk for sudden death, proper care could be lifesaving. In 2020, the Journal of the American Heart Association published a consensus guideline from worldwide experts, for cardiologists caring for DM1 patients [131]. One of the main points was that cardiac manifestations can precede other aspects of the disease including skeletal muscle phenotypes such as weakness and myotonia. Another key point was that cardiac manifestations can occur in children and young patients. Manifestations to be aware of include: (1) atrial arrhythmias; (2) embolic events (possibly secondary to increased risk due to atrial arrhythmias; (3) progressive heart block; (4) ventricular arrhythmias; (5) heart failure; and (6) cardiomyopathy. 

The guidelines recommend:

(1) Baseline 12 lead ECGs and annual ECGs in asymptomatic individuals;
(2) Cardiac imaging (either Echocardiography or CMR) at baseline and every one to five years;
(3) Ambulatory monitoring (e.g., Holter monitoring) to detect asymptomatic arrhythmias;
(4) Invasive electrophysiology if other tests show high risk conduction defects;
(5) Patient and family education for signs and symptoms of heart disease and coronary artery disease (e.g., angina);
(6) Awareness of risk for hyperlipidemia, metabolic syndrome (role of exercise?);
(7) Treatment of atrial fibrillation;
(8) Control of blood pressure;
(9) Heart failure treatment in patients with LV ejection fraction <50%;
(10) Pacemakers or ICD (implantable cardioverter-defibrillators) for patients at risk for sudden death;
(11) Cardiac resynchronization therapy for select patients.

## 10. Conclusions

Ever since the first description of DM1 by Steinert, it was apparent that cardiac pathology in DM1 is very common and can have devastating consequences. Cardiac conduction abnormalities are the primary manifestations with over 75% of DM1 patients developing varying degrees of dysrhythmias during their lifetime. Though most of the focus is on atrioventricular blocks and prolongation of PR intervals, just about any arrhythmia is seen in DM1. One common class of arrhythmias that probably needs more attention, are atrial arrhythmias. These are very common in DM1 (up to 30% of cases) and studies have associated atrial arrhythmias with increased risk of sudden death. Sudden death is the second most common cause of death in DM1. Very little is known about the cause of sudden death, and though it is more common in patients with more severe arrhythmias, it is not unusual to see sudden death in individuals with no prior cardiac history.

RNA toxicity underlies the pathology in DM1. It leads to a multitude of RNA splicing defects that may drive the pathology through effects on cardiac channels and structural proteins. Further research is necessary to understand the role of any one of them in the context of RNA toxicity. Histologic studies of the heart in DM1 have been scarce. However, common findings are interstitial fibrosis and fatty infiltration of the myocardium. Recent studies using CMR show promise in detecting these changes and suggest that the myofibroblast driven changes may be very common and precede clinical manifestations and identify patients at risk

Multiple mouse models of RNA toxicity have provided strong evidence for the role of RNA toxicity in cardiac pathology in DM1. Though, not all aspects of DM1 cardiac pathology have been reported in most of the models, with the DM200 mouse model likely the most comprehensive model thus far; the entirety of data suggests that RNA toxicity accounts for most if not all of the molecular mechanism underlying cardiac pathology. Importantly, the inducible/reversible models have shown the potential for treating cardiac pathology by targeting the degradation or silencing the expression of the toxic RNA.

Current treatment approaches for cardiac disease in DM1 fall within standard paradigms of cardiology practice. A number of approaches are being investigated to treat DM1, including small molecules, gene therapy, antisense oligonucleotides (ASOs), CRISPR/Cas9 mediated deletion of (CTG) repeats, and cell-based therapies [132]. Many companies are developing ASO based approaches to treat DM1. Our recent work showed that ASOs could be a possible option for treating cardiac manifestations of DM1 [111]. Further insights from ongoing work in mouse models is likely to identify new targets and biomarkers that may prove useful in clinical trials and treatment of heart disease in DM1.

## Figures and Tables

**Table 1:** Currently Existing Mouse models of RNA toxicity in DM1. Tabulation of relevant clinical and molecular phenotypes in various mouse models of RNA toxicity; + (present); − (absent). Abbreviations: NR, not reported; NA, not applicable.

Model	Prolonged PR -ECG	Afib/Afl on ECG	Progressive Conduction Defects	RNA Splicing Defects	Sudden Death	Cardiac Fibrosis	Reference
DM1 Patients	+	+	+	+	+	+	
DM5	+	+	+	NR	+	NR	[66]
DM200	+	+	+	+	+	+	[111]
HSALR Sk. Muscle specific	−; NA	−; NA	−; NA	−; NA	−; NA	−; NA	[115]
DMSXL	−	−	−	few	−	−	[117]
LC15	−	−	−	+	−	−	[114]
TREDT960I	−	−	−	+	−	NR	[113]

## Data Availability

The data that support the findings of this study are available from the corresponding author upon reasonable request.
